# Analysis of 4 Cases of Left Bundle Branch Block With Ventricular Preexcitation

**DOI:** 10.1111/anec.70091

**Published:** 2025-06-01

**Authors:** M. M. Li Yi, B. M. Li Xingjie

**Affiliations:** ^1^ Hematology Department Jining no. 1 People's Hospital Jining Shandong China; ^2^ Electrocardiogram Room Jining no. 1 People's Hospital Jining Shandong China

**Keywords:** arrhythmia, electrocardiogram, left bundle branch block, WPW

## Abstract

This paper reports 4 cases of ventricular preexcitation (WPW) accompanied by left bundle branch block (LBBB). WPW appeared intermittently in all cases: Case 1 involved a right‐sided accessory pathway, while Cases 2–4 involved left‐sided accessory pathways. The electrocardiographic characteristics of both right‐sided and left‐sided accessory pathways coexisting with LBBB are discussed.

Chiale P.A. et al. (Chiale and Elizari 2012) noted that WPW with LBBB is rare. We hereby report 4 cases encountered in our practice.

## 1 Discussion

LBBB and WPW are distinct arrhythmias characterized by wide QRS complexes. When coexisting, their ECG interpretation becomes challenging. Pick (Pick and Fisch 1958) reported a 0.24% coexistence rate of bundle branch block (BBB) and WPW in 100,000 ECGs. Since right BBB (RBBB) is twice as common as LBBB (Surawicz and Knilans 2008), the estimated prevalence of LBBB + WPW is – 0.08% (Pick and Fisch 1958).

Pick (Pick and Fisch 1958) proposed:

1. WPW can coexist with RBBB or LBBB.
2. If the accessory pathway and BBB are contralateral, both features are discernible.
3. Ipsilateral pathways pre‐excite the blocked ventricle, masking BBB.
4. Bilateral pathways may obscure BBB.

The four patients in this study did not undergo intracardiac electrophysiological studies, and the localization of the atrioventricular accessory pathways could only be determined based on the direction of the Δ waves (Surawicz and Knilans 2008) in the surface electrocardiogram (see Table 1).

**TABLE 1:** Clinical profiles and ECG characteristics of Cases 1–4.

Case no.	Gender	Age	Clinical diagnosis	Accessory pathway localization (based on Δ wave direction)	WPW presentation	LBBB presentation	Characteristics of WPW + LBBB	QRS features in OAVRT	Other findings
Case 1	Male	22	Hypertrophic Obstructive Cardiomyopathy	Right lateral wall	Intermittent	Persistent	Neither masks the other		
Case 2	Female	50	① Type 2 Diabetes; ② Hypertension; ③ Coronary Artery Disease	Left posterior septum	Intermittent (Figure 2B)	Intermittent (Figure 2A)	WPW masks LBBB	LBBB‐typ	
Case 3	Male	84	Coronary Artery Disease	Left anterior wall	Intermittent	Persistent	WPW masks LBBB		
Case 4	Male	73	① Type 2 Diabetic Nephropathy; ② Hypertension; ③ Coronary Artery Disease	Left anterior wall	① Intermittent; ② Complete (Figure 4A); ③ Incomplete (Figure 4B)	Persistent	① Complete WPW obscures LBBB (Figure 4A); Incomplete WPW masks LBBB (Figure 4B)		Intermittent First‐Degree AVB (Figure 4A)

Abbreviations: AVB, atrioventricular block; OAVRT, orthodromic atrioventricular reentrant tachycardia.

**FIGURE 1:**
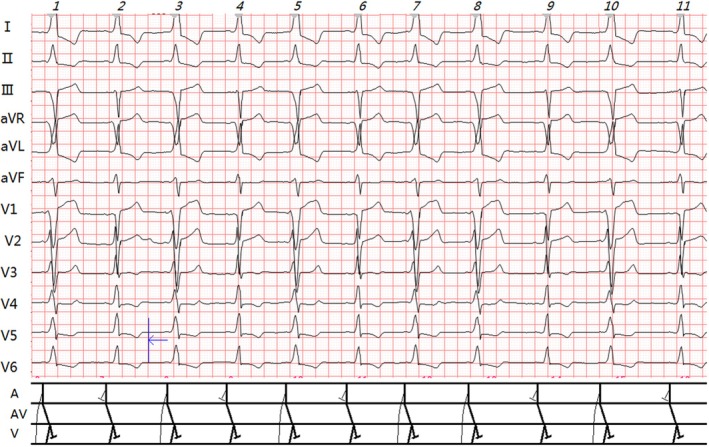
Case 1's standard ECG (calibration: 1 mV = 2.5 mm). Slanted numbers above the figure denote QRS complex sequence. In the ladder diagram, thin arcs represent accessory pathway conduction, and thick lines indicate normal pathway conduction (same for subsequent figures). Additional details are provided in the text. Clinical summary of Case 1 (Table 1). ECG Analysis (Figure 1): Sinus rhythm (64 bpm). Two types of QRS complexes: 1. Isolated LBBB (R2, 4, 6, 9, 11): PR 0.14 s; no delta wave; broad R waves in leads I, aVL, V5, V6; QS pattern in V1; QRS duration 0.16 s; PJ interval 0.30s. 2. WPW + LBBB (R1, 3, 5, 7, 8, 10): PR 0.08 s; Δ wave present; QRS duration 0.22 s; PJ 0.30s; rS pattern in V1–V3 (r/S < 1, rV1 blunted); R/S transition at V4; R/S > 1 in I, aVL. Diagnosis: ① Sinus rhythm; ② LBBB; ③ Intermittent WPW (right‐sided pathway).

Case 1 (right‐sided pathway + LBBB): Due to contralateral pathways, both features remained visible (Figures 1 and 5B). PJ intervals were 0.30s.

**FIGURE 2:**
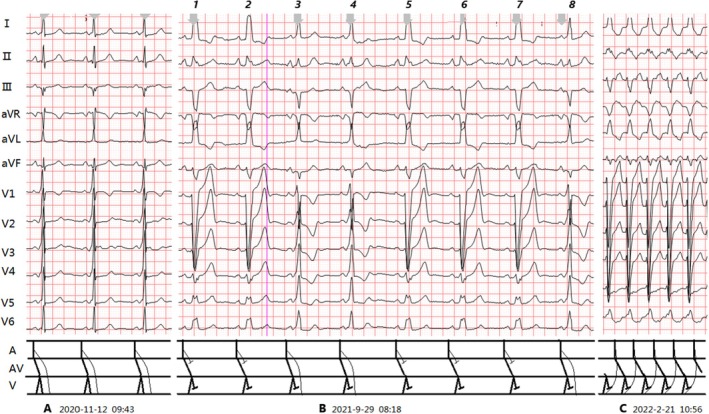
(A–C) (Case 2): Clinical summary of Case 2 (Table 1). ECG analysis: (A) Sinus rhythm (83 bpm); typical WPW without LBBB (Figure 5A): PR 0.11 s, delta wave, Rs/R pattern in V1‐V6; QRS duration 0.14 s; PJ 0.26 s. (B) Sinus rhythm (63 bpm). Two QRS types: 1. Isolated LBBB (R1, 2, 5–7): PR 0.16 s; no Δ wave; broad R waves in I, aVL, V5, V6; rS in V1; QRS 0.16 s; PJ 0.32 s. 2. WPW + LBBB (R3, 4, 8): PR 0.11 s; Δ wave present; V1 shifts from Rs (A) to rS due to LBBB; R pattern in V2‐V6; QRS 0.14 s; PJ 0.26 s. (C) OAVRT (166 bpm); QRS morphology similar to LBBB in (B); RP^−^ interval 0.16 s, *P* ^−^R 0.20s. Diagnosis: ① Sinus rhythm; ② Intermittent LBBB; ③ Intermittent WPW (left‐sided pathway); ④ OAVRT.

**FIGURE 3:**
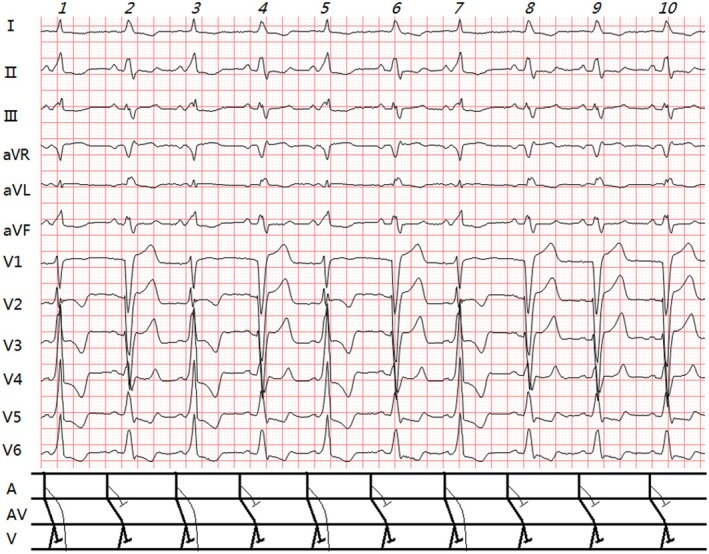
(Case 3): Clinical summary of Case 3 (Table 1). ECG analysis: Sinus rhythm (71 bpm). Two QRS types: 1. Isolated LBBB (R2, 4, 6, 8–10): PR 0.16 s; QRS 0.16 s; QS in V1; broad R waves in I, aVL, V5, V6; PJ 0.32 s. 2. WPW + LBBB (R1, 3, 5, 7):PR 0.12 s; Δ wave present; QRS 0.17 s; PJ 0.29 s; rS in V1, Rs/R in V2‐V6. Diagnosis: ① Sinus rhythm; ② LBBB; ③ Intermittent WPW (left‐sided pathway).

**FIGURE 4:**
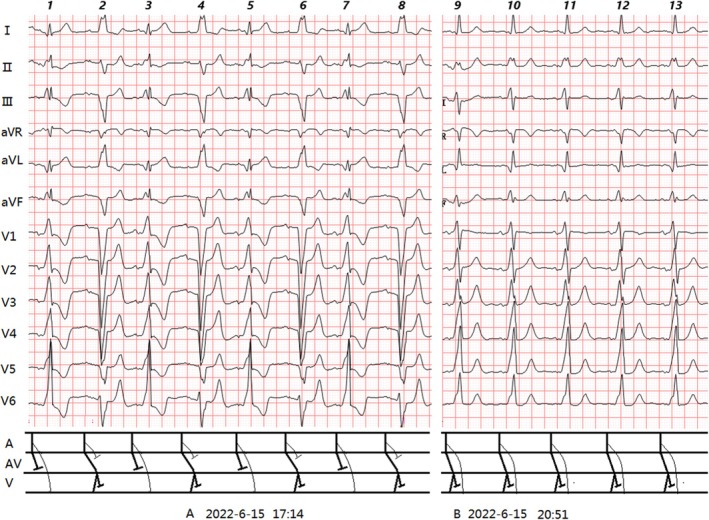
(A–B) (Case 4): Clinical summary of Case 4 (Table 1). ECG Analysis: (A):Sinus rhythm (79 bpm). Two QRS types: 1. Isolated LBBB (R2, 4, 6, 8): PR 0.24 s; QRS 0.16 s; PJ 0.40s. 2. Complete WPW (R1, 3, 5, 7): PR 0.12 s;Δ wave present; QRS 0.18 s; PJ 0.30s; R/Rs pattern in V1‐V6. (B) WPW + LBBB (R9–13): PR 0.12 s; Δ wave present; QRS 0.18 s; PJ 0.30s; hybrid QRS morphology (notable in I, III, V1, V2); rS in V1, Rs/R in V2‐V6. Diagnosis: ① Sinus rhythm; ② Intermittent first‐degree AVB; ③ LBBB; ④ Intermittent WPW (left‐sided pathway).

**FIGURE 5:**
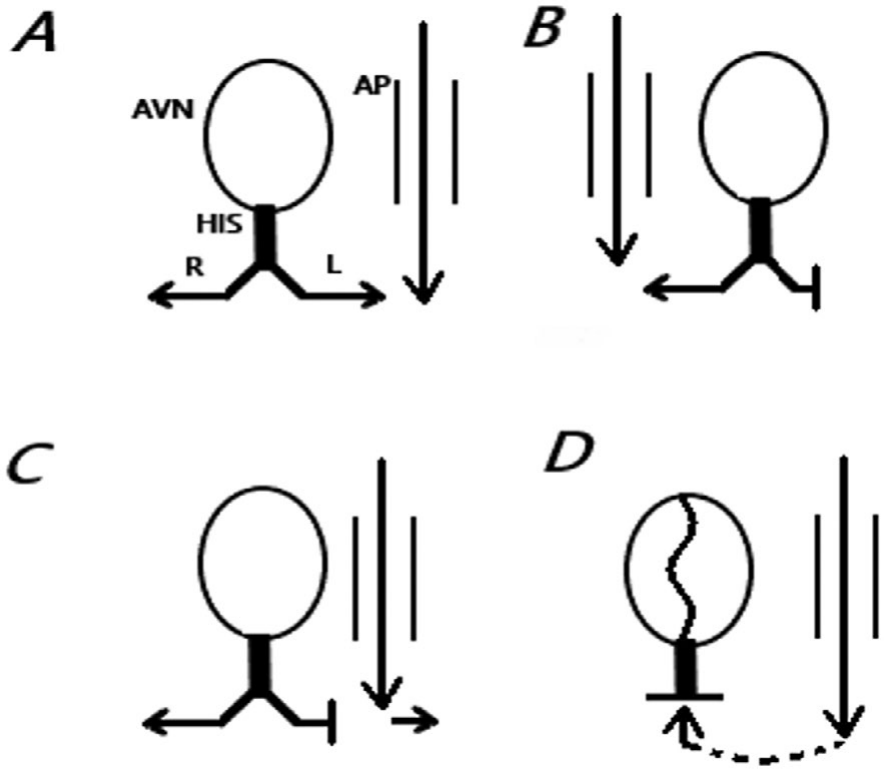
Scenarios of LBBB with WPW. (A) WPW without bundle branch block; (B) Right‐sided pathway with LBBB: Neither masks the other; (C) Left‐sided pathway with LBBB: Accessory pathway pre‐excites the delayed LBBB region, masking LBBB; (D) Left‐sided pathway with first‐degree AVB: Complete WPW obscures LBBB due to retrograde block in the normal pathway.

Case 2–4 involves a patient with a left‐sided accessory pathway and LBBB. Since the accessory pathway and the BBB are on the same side, part of the left ventricular myocardium that was originally activated with a delay due to the LBBB is pre‐activated by the impulse conducted through the accessory pathway. Therefore, the waveform of the LBBB is often masked by the WPW, and the P‐J interval is shortened. When the combination of LBBB and WPW appears continuously, because the waveforms of both are atypical, it is extremely easy to lead to missed diagnosis or misdiagnosis (see Figures 2, 3, 4 and Figure 5C). Only when one of them appears intermittently can the other show a typical manifestation. Therefore, it is necessary to make full use of the electrocardiographic information of pure LBBB or WPW revealed by intermittent WPW, intermittent LBBB, OAVRT, or junctional activation for analysis and identification.

Case 4 (Figure 4A): First‐degree AVB (PR 0.24 s) delayed normal pathway conduction, allowing exclusive accessory pathway activation (Figure 5D). LBBB was entirely masked. Complete WPW and LBBB alternated, mimicking RBBB‐LBBB alternation. Fusion waves emerged only after AVB resolution (Figure 4B).

In Ebstein's anomaly, RBBB often coexists with WPW. Iturralde et al. found that 94% of patients exhibited RBBB post‐ablation (Chiale and Elizari 2012). Whether LBBB and WPW share similar associations remains unclear; our cases likely reflect random coincidence.

## Author Contributions

The author takes full responsibility for this article.

## Conflicts of Interest

The authors declare no conflicts of interest.

## Data Availability

The data that support the findings of this study are available from the corresponding author upon reasonable request.
